# Why Does the Giant Panda Eat Bamboo? A Comparative Analysis of Appetite-Reward-Related Genes among Mammals

**DOI:** 10.1371/journal.pone.0022602

**Published:** 2011-07-27

**Authors:** Ke Jin, Chenyi Xue, Xiaoli Wu, Jinyi Qian, Yong Zhu, Zhen Yang, Takahiro Yonezawa, M. James C. Crabbe, Ying Cao, Masami Hasegawa, Yang Zhong, Yufang Zheng

**Affiliations:** 1 School of Life Sciences, Fudan University, Shanghai, China; 2 Faculty of Creative Arts, Technologies and Science, Institute of Applied Natural Sciences, University of Bedfordshire, Luton, United Kingdom; 3 Institute of Statistical Mathematics, Tokyo, Japan; 4 Institute of Biodiversity Science and Institute of High Altitude Medicine, Tibet University, Lhasa, China; Ecole Normale Supérieure de Lyon, France

## Abstract

**Background:**

The giant panda has an interesting bamboo diet unlike the other species in the order of Carnivora. The umami taste receptor gene *T1R1* has been identified as a pseudogene during its genome sequencing project and confirmed using a different giant panda sample. The estimated mutation time for this gene is about 4.2 Myr. Such mutation coincided with the giant panda's dietary change and also reinforced its herbivorous life style. However, as this gene is preserved in herbivores such as cow and horse, we need to look for other reasons behind the giant panda's diet switch.

**Methodology/Principal Findings:**

Since taste is part of the reward properties of food related to its energy and nutrition contents, we did a systematic analysis on those genes involved in the appetite-reward system for the giant panda. We extracted the giant panda sequence information for those genes and compared with the human sequence first and then with seven other species including chimpanzee, mouse, rat, dog, cat, horse, and cow. Orthologs in panda were further analyzed based on the coding region, Kozak consensus sequence, and potential microRNA binding of those genes.

**Conclusions/Significance:**

Our results revealed an interesting dopamine metabolic involvement in the panda's food choice. This finding suggests a new direction for molecular evolution studies behind the panda's dietary switch.

## Introduction

Biodiversity is showing up not only how different animals appear, but also on how different their diets are. Some species have unique diets and it is interesting to look for the evolutionary reasons behind such natural selection. One such example is the giant panda. As one species of Ursidae (the bear family), Carnivora, the giant panda (*Ailuropoda melanoleuca*) has a very special bamboo diet. The bears are normally carnivores to omnivores. For example, the polar bears survive solely on meat and fat, while the black bears are more or less omnivores. About 7 Myr ago, the ancient giant panda was still omnivorous [1]. About 2–2.4 Myr ago, they become herbivores as soft bamboo shoots, stems, and leaves became their major food source [1]. The giant panda also developed an enlarged radial sesamoid that functions as a thumb to grab bamboo [2], [3], [4]. The bamboos have relatively low energy and nutrition compare to meat and fruits. Therefore, the average giant panda needs to consume up to 6% of its body weight of bamboo in dry matter per day to survive [5], [6], [7]. However, the giant panda's digestive system is still more fit for a meat diet than bamboo as they can use less than 20% of the bamboo they ingest [7], [8], and both its gut anatomy and microbiome have not yet adapted to degrade those bamboo fibers [9]. Therefore, what is the driving force behind the panda's diet switch is still a question for evolutionary biologists.

Recently, two papers reported that the *T1R1* gene in the giant panda turned into a pseudogene due to two frame-shifting mutations in exon 3 and 6, respectively [8], [10]. T1R1 is part of the T1R1/T1R3 heterodimer receptor that mediates umami taste. Thus, this lost-of-function on the *T1R1* gene in the giant panda may contribute to the panda's food choice [8], [10]. This is a breakthrough on how the giant panda has become the species it is. However, there are some missing links between the lost meat taste and the bamboo diet of the giant panda. First of all, taste is not the only environmental cue that can affect animals' eating behaviours. To survive, energy and nutrition properties of food can highly influence animals' food choices, while smell and taste are associated with those properties [11], [12]. Therefore, even without the ability to taste meat, giant pandas can still choose meat as their main diet since meat contains much higher energy and nutrition than bamboo. Meat is also available in the giant panda's habitats as other carnivores such as wolf and dog share the same area. Secondly, the estimated mutation time for the *T1R1* gene is probably 4.2 Myr for the giant panda [10]. The fossil evidence showed that the giant panda started eating bamboo at least 7 Myr ago, and at about 2.0–2.4 Myr ago they probably had already completed their dietary switch [1]. Therefore, the pseudogenization of *T1R1* is probably the result of, not the reason for, its dietary change. Thirdly, the *T1R1* gene is intact in some herbivores such as cow and horse [10], which indicates that the taste is probably not the only reason for an animal's food choices. Therefore, we need to look beyond taste to understand the driving force for the panda's diet.

To answer such a question, we also need to look on how and why taste can trigger eating behaviour. In the wild, animals are normally attracted by sweet and umami (savory) stimuli. The umami taste is excited by L-glutamate, which is abundant in meat [13], [14]. The sweet taste is excited by saccharides [13], [14]. Both chemicals indicate the food is high in nutrition and energy, which is crucial for an animal's survival in the wild. Therefore, such a taste is related with the physical rewarding properties of food that in turn further affect the feeding behaviour of the animal. In other words, both the cues (such as umami taste) and properties (such as rich in nutrition and energy) of the food are stored in memory to guide future behaviour, such as to orient the animal back to the source of food [11], [12]. To the giant panda, without being able to taste the meat, the nutrition and energy properties of food should play a more critical role in driving its feeding behaviour. However, the bamboo diet does not fit to this hypothesis, suggesting that there might be something special in the appetite-reward system for the giant panda. To understand such bias, we looked deep in the appetite-reward circuitry and did a comprehensive analysis on genes involved in this appetite-reward system based on the giant panda's draft genome published last year [8]. Our results revealed a complex genetic background and an interesting dopamine metabolic involvement behind the taste for the giant panda's bamboo diet.

## Results and Discussion

The tendency to engage in or maintain feeding behaviour is potently influenced by the flavour of food, the gut reaction to the components of food, and the reward pathways in the brain [11], [15], [16], [17], [18]. Animal experiments have shown that both opioid and dopamine are related to appetite-reward circuitry in food intake behaviour [17], [18]. Based on those studies in human and rodents [11], [15], [16], [17], [18], we undertook a comprehensive analysis on about 166 major genes (see a list of gene full name and Ensembl number in table S1) in the panda that are involved in the appetite and food reward system for their coding regions and untranslated regions, especially the 3′UTR and ATG region. We also constructed an online database for analysis on those genes, which can be accessed at http://idm.fudan.edu.cn/Apanda/ (Fig S3).

### 1. Analysis on coding region

**Figure 1:**
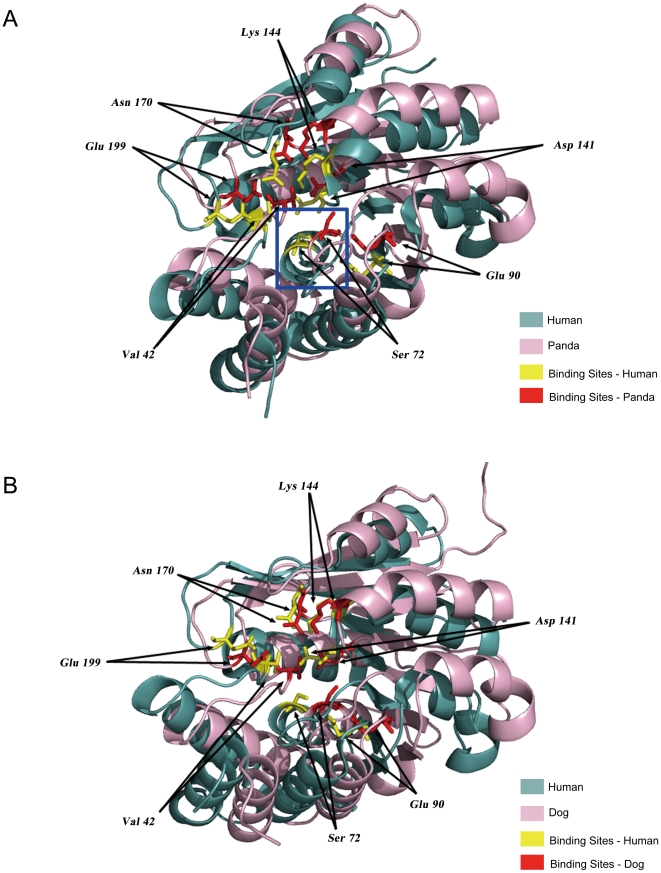
Panda and dog COMT protein structural simulation. The simulated panda or dog proteins were indicated with pink color for backbone and red color for special amino acid residues. Human proteins were indicated with blue color for backbone and yellow color for special amino acid residues. The side chain of COMT catecholamine substrate binding sites (Lys_144_, Asn_170_, Glu_199_) and SAM binding sites (Val_42_, Ser_72_, Glu_90_, Asp_141_) were shown in ball and stick model. (**A**) The simulated panda COMT structure compared with human COMT. The α4 helix in blue square has turned into a loop in panda. (**B**) The simulated dog COMT structure compared with human COMT.

**Figure 2:**
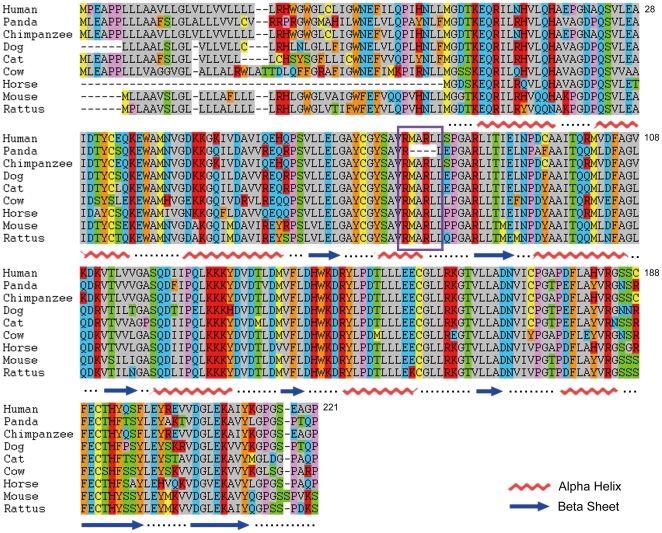
Sequence alignment of COMT from all nine species. The number for amino acids is based on human soluble COMT. The conserved amino acids were highlighted. The regions for alpha helix and beta sheet were marked at the bottom of the alignment (wave for alpha helix and arrow for beta sheet). The region with missing amino acids in panda is highlighted with purple square box.

We first screened out the coding region of those selected genes. To look for possible unique sites for the giant panda, we first compared the sequences of giant panda with human (*Homo sapiens*). Interestingly, most of those genes are highly conserved which is probably due to the biological importance of food intake for survival. Therefore, to look for the possible structural changes of those proteins based on the panda's sequence, we then screened those genes based on their similarity to human orthologs. The screening was performed following two criteria: (i)>75% consensus sequence between panda and human; and (ii) with human protein structures reported in PDB (sequence coverage >75%). Six genes of the panda were screened out (Table 1, *COMT, MAOA, MAOB, LEP, ALDH2, PNMT*) and structural simulations for those six proteins were done based on the structure of human orthologs by Modeller 9v8 software [19]. Our results revealed that most of them had no major changes in the panda (Figure S1A for MAOA), except one, COMT (catechol-o-methyltransferase), which had a significant change in its substrate/cofactor SAM (S-adenosyl-L-methionine) binding domain (Figure 1A). COMT is one of enzymes that inactive catecholamine neurotransmitters, such as dopamine, epinephrine, and norepinephrine [20], [21]. The enzyme introduces a methyl group to the catecholamine, which is donated by the CE methyl group on the methionine portion of SAM [22], [23]. The methionine portion of SAM is fixed to proper position by hydrogen bonds with several crucial residues in α3 (V42), α4 (S72), and β4 (D141) in human COMT [23], [24]. Although those crucial amino acids were conserved in panda, the α4 helix is lost due to a four amino acids deletion mutation in panda COMT and this part became a more flexible loop in the panda's COMT structure (Figure 1A, blue square & Figure 2 for alignment). This flexible loop may very likely affect the conformation of the methionine portion of SAM and the methyl transfer between the CE methyl group of SAM and catecholamines. Therefore, such structural variation of the panda's COMT suggests a possible relatively slow metabolic turnover rate of catecholamine neurotransmitters by COMT in panda. It has been shown that COMT-deficient male mice had elevated dopamine levels in the frontal cortex [25], and elevated dopamine could enhance motivation for food in mice [16]. In humans, several mutations on *COMT* were associated with eating disorders [26], [27] and obsessive-compulsive disorder in men [28]. Therefore, it is very likely that the potential lower activity of COMT in panda is related to its special feeding behavior.

**Table 1:** Six genes were selected out based on protein structure and sequence consensus percentages.

Protein Name	PDB Id	Coverage#	Percentage#	Consensus*	Percentage*
COMT	3BWM	214/271	78.97%	206/271	76.01%
LEP	1AX8	145/167	86.83%	136/167	81.44%
ALDH2	1O05	500/517	96.71%	479/517	92.65%
MAOA	2BXR	527/527	100.00%	454/527	86.15%
PNMT	1YZ3	282/282	100.00%	247/282	87.59%
MAOB	1GOS	520/520	100.00%	475/520	91.35%

#: The sequence coverage and their percentage between human structure in PDB and human reference proteins.

*: The sequence consensus and their percentage between panda and human proteins.

To further check the evolutional effect on those six genes, we then checked the orthologs of seven other species from Ensembl, including chimpanzee (*Pan troglodytes*), mouse (*Mus musculus*), rat (*Rattus norvegicus*), dog (*Canis lupus familiaris*), cat (*Felis catus*), horse (*Equus caballus*), and cow (*Bos taurus*). Those species have various diets as indicated in Li's paper [8]. For example, cat and dog are carnivores, horse and cow are herbivores, and the others are omnivores. We performed the structure simulation for the six proteins from those species and compared to human orthologs. In general, the structures are highly conserved and very similar to human orthologs. As for COMT, the substrate binding domains of other species were very similar to human COMT even though there is some small shift on the whole structure (Figure 1B for dog COMT and others in our database). The sequence for the α4 helix is totally conserved in all species we checked except panda (Figure 2).

### 2. Analysis of Kozak motif at the ATG starting site

Besides the coding region, a gene's expression level is highly regulated by multiple ways, including transcription regulation and post-transcription regulation. The 5′UTR region is critical in transcription regulation as there are several mechanisms involved, such as transcription factors binding to the promoter region to modify the efficiency of transcription, the start of a protein-coding sequence that affects the polymerase complex binding to DNA, etc. Since the transcription factor binding sites are complex, as there are both enhancing and depressing effects by transcription factors, it is inconclusive to analyze them based solely on DNA sequence information. Therefore, we decided to focus on the starting site of those genes, which is the Kozak consensus sequence.

The Kozak consensus sequence is a sequence that occurs on eukaryotic mRNA and has the consensus gccRccAUGG in vertebrates [29], [30], [31]. Some nucleotides in this sequence are more important than others: the AUG is essential since it is the actual initiation codon. For a ‘strong’ consensus, the nucleotides at positions +4 and -3 must both match the consensus. An ‘adequate’ consensus has only 1 of these sites, while a ‘weak’ consensus has neither. Although a G in the -6 position and CC at -1 and -2 contribute to the overall strength, they are less essential in initiating translation [29], [30]. Therefore, to compare the strength between human and panda Kozak sequence, we used AUG, -3, and +4 positions as criteria (Table 2 and Table S2). To eliminate the effects of sequencing errors, we first removed the genes without the AUG initiation codon from the 166 genes targeted. There are 78 genes removed and the 88 genes left were divided into two parts, (i) 7 genes present the different pattern at -3 or +4 position or both positions (Table 2, *MC4R, OPRD1, COMT, ADRA1D, GRIA3, HTR3E, GRM7*), and (ii) 81 genes have the same pattern at -3 and +4 position (Table S2). Four of those seven genes (*COMT, MC4R, OPRD1, GRM7*) have weaker Kozak consensus sequence in the giant panda that probably can also cause a lower expression of the respective gene (Table 2, gene name in bold). Interestingly, *COMT* is also included in this list and such a weaker Kozak motif probably can further enhance the effect of its coding region.

**Table 2:** Seven genes with different Kozak sequence between panda and human.

	Panda				Human			
Gene name	ATG	G+4	R-3	Strength	ATG	G+4	R-3	Strength
***COMT***	y			W	y		y	A
***MC4R***	y		y	A	y	y	y	S
***OPRD1***	y	y		A	y	y	y	S
***GRM7***	y		y	A	y	y	y	S
*ADRA1D*	y	y	y	S	y		y	A
*GRIA3*	y	y	y	S	y	y		A
*HTR3E*	y	y	y	S	y			W

A “y” is labeled to indicate that the nucleotides match the consensus sequence. The strength of Kozak motif is labeled with “S” for strong, “A” for adequate, and “W” for weak. Those genes have a weaker Kozak sequence in panda were marked with bold characters.

We also checked the Kozak motif of those seven genes in other species and all species have its own expression pattern for those seven genes. Such variations and diversity may somehow relate to the complex genomic background and specific characters of each species (Table S3).

### 3. Analysis on potential MicroRNA binding on 3′UTR

**Figure 3:**
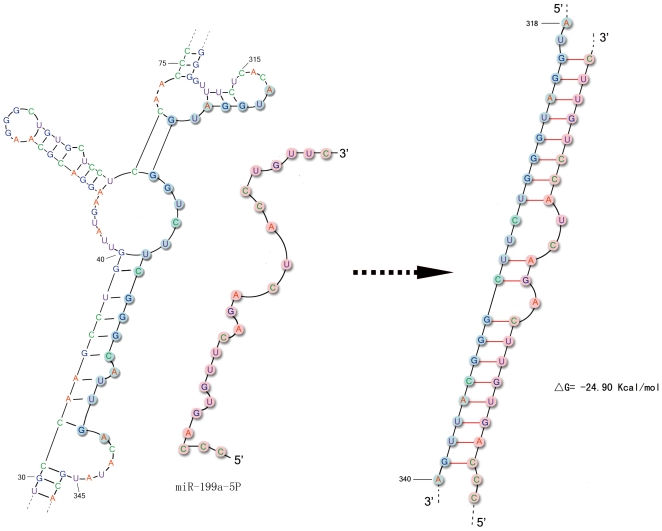
Part of the panda *COMT* 3′UTR and predicted miRNA-199a-5p binding. *COMT* 3′UTR secondary structure was predicted by Mfold and part of it was shown on the left. The possible miRNA-199a-5p target with panda *COMT* gene and the calculated free energy was shown on the right side.

Post-transcription regulation has also been recognized more and more importantly in regulating gene expression level. After being produced, the stability and distribution of the different transcripts can be regulated by several important mechanisms such as RNA interference (RNAi) and RNA binding protein. Two types of small RNA molecules, microRNA (miRNA) and small interfering RNA (siRNA) are central to RNAi. MiRNAs are post-transcriptional regulators that bind to complementary sequences on target messenger RNA transcripts (mRNAs), usually resulting in translational repression and gene silencing [32]. MiRNAs are well conserved in eukaryotic organisms and are thought to be a vital and evolutionary ancient component of genetic regulation [33], [34]. Therefore, we focused on those genes that have been screened out by the first two methods to predict potential miRNA binding. There are 6 genes screened out by the first method and 7 genes screened out by the second method, and *COMT* is screened out by both methods. Therefore, there are total 12 genes checked to predict potential miRNA binding. 9 out of 12 genes have at least one computational predicted miRNA (Table S4). Two of those genes, *COMT* and *ADRA1D* (adrenergic receptor, alpha-1D), were predicted to be regulated by miRNA in panda but not in human. MiR-30C was computationally predicted to bind the 3′UTR of panda *ADRA1D* gene and miR-199a-5p to the 3′UTR of panda *COMT* gene (Figure 3 & Figure S2). The adrenergic receptors are G protein-coupled receptors that are targets of the catecholamine, especially norepinephrine and epinephrine. The predicted miRNA regulation may down-regulate the COMT and ADRA1D's protein expression level in the giant panda and enhance the effects of deficient catecholamine metabolism.

### Conclusion

The giant panda's dietary switch to bamboo is unique and interesting. The pseudogenization of its umami receptor gene *T1R1* is coincident with such bamboo dietary switch. But there are probably other factors involved in such a switch. To look beyond taste and the microbiome in panda that has the potential affect on this dietary switch, we studied the giant panda's appetite-reward system.

Our comprehensive sequence analysis on the giant panda's appetite-reward systems indicated that the panda probably has some defects in its catecholamine metabolic pathways, which thus affect its food choices. This prediction provides a new insight for better understanding the giant panda's specific characteristics. However, eating behaviour is an extremely complex process. Besides the homeostatic regulation by the hypothalamus, the cortical-limbic system is also involved in regulating response to environmental conditions and stimuli such as the smell and taste of food [1], [3], [8]. In addition, too much or too little dopamine both has profound effects on feeding behaviours [2]. It would be interesting to carry out detailed biochemical assay on those enzymes, such as COMT, that are involved in appetite-reward circuitry and compare them to other family of bears and other species, e.g. dog.

Based on our analysis, the dopamine metabolic system is probably not that competent in the giant panda. Recently, it has been shown that dopamine is essential for stimulus-reward learning behaviour [35]. Therefore, such deficient dopamine metabolism in the giant panda may have some profound effect on its reward-cue directed behaviour. It is possible that some ingredient in the bamboo may able to help the catecholamine, especially dopamine, metabolism in panda. Such an ingredient may able to stimulate the appetite-reward circuitry in the giant panda and play certain role in setting up its bamboo diet. Further analysis on bamboo chemical components and their effects on nerve system will be needed.

Even then, the initial evolutionary reason for the giant panda to choose such a diet is still a mystery. Further investigation on the carnivore catecholamine metabolism and its effect on food choice will be essential. In addition, comparative genomics of the bear family, Ursidae, as well as *Ailurus fulgens* (red panda), which has a similar diet to the giant panda, will be helpful to understand carnivore food choices.

## Materials and Methods

The genome sequence of the Giant Panda was downloaded from BGI (Beijing Genomics Institute, http://panda.genomics.org.cn/page/panda/index.jsp) and compared with the data from Ensembl (Ensembl Genome Browser). The high quality genome and reference sequence of eight other species were also retrieved from Ensembl, which include human (*Homo sapiens*), chimpanzee (*Pan troglodytes*), mouse (*Mus musculus*), rat (*Rattus norvegicus*), dog (*Canis lupus familiaris*), cat (*Felis catus*), horse (*Equus caballus*), and cow (*Bos taurus*). The 166 targeted genes are selected based on the studies about appetite and food intake behaviour [11], [15], [16], [17], [18]. Orthologs were determined and mapped to Ensembl gene identifiers using BioMart [36], [37]. Please see a list of gene name, full name, and Ensembl number in our database and supplementary table S1.

### Panda protein 3D-structure simulation

To see how the different amino acids in a panda protein would affect its structure, we carried out the following simulation analysis. First, we filtered the datasets containing 166 proteins by two criteria (i) the sequence consensus between the protein in panda and its ortholog in human is above 75%; and (ii) the gene's ortholog in human must have 3D-structure with sequence coverage above 75%. With these two criteria, we filtered out six proteins (Table 1) and simulated panda proteins using Modeller 9v8 [19] to compare with their human orthologs. Then we also performed structure simulation of those six proteins for the other seven species.

For the COMT protein, we performed structure simulation based on the 3D-structure of human COMT protein (PDB ID: 3BWM). We obtained the simulated 3D-structure of COMT protein in panda and aligned with human COMT to visualize the difference (Figure 1A). The dog and human COMT structure alignment was presented in Figure 1B.

By using the strategy described above, we performed structure simulation in panda on the 3D-structure of human MAOA protein (PDB ID: 2BXR) and aligned with its human ortholog. These two structures were presented in Figure S1A. The simulated dog MAOA aligned with human ortholog were presented in Figure S1B.The simulated structures for other species are stored in our database.

For the other four proteins (LEP, ALDH2, PNMT, MAOB), the sequence between human and panda are highly conserved (Table 1). Similar methods were applied and all simulation structures are stored in our database (http://idm.fudan.edu.cn/Apanda/) (Figure S3).

### Sequence alignment of COMT

The alignment of COMT from nine species were performed by MAFFT 6.850 [38], which has an auto mode allowing the program to choose proper parameters for each sequence sets.

### Kozak motif analysis

For all previous described 166 genes, we carried out Kozak motif analysis to evaluate the Kozak consensus of each gene within nine species. For all genes, we downloaded the DNA genome sequences from Ensembl. Based on the annotation of the released genome sequences on Ensembl, we identified where the coding sequence starts. To eliminate the effects of sequencing errors, we first removed 78 panda genes without the AUG initiation codon from the 166 genes targeted. Then we extracted the 30 bp nucleotide sequences of each gene at position −25∼+5 based on the annotation for those 88 panda genes left. If the sequence included the nucleotides without masked characters, we extracted the Kozak sequence directly from them. Otherwise, we manually identified a 30 bp sequence between position −25∼+5 by using the NCBI sequence viewer.

### Prediction of 3′ UTR in Panda

The genes screened out by the first two methods were combined here to check for potential miRNA binding. Since COMT was screened out by both methods, there are 12 genes (6+7−1 = 12) checked here for potential miRNA binding. As the 5′- and 3′- untranslated regions (UTR) for genes in the panda genome are without annotation, 4 kb sequences downstream of the coding sequence (CDS) of the above 12 genes were extracted for 3′UTR analysis. We used the Polyadq program [39] with default settings to predict the polyadenylation site (polyA) (http://rulai.cshl.org/tools/polyadq/polyadq_form.html), which is a major signal for transcription termination. This sequence was also compared with the multiple sequence alignment file of the 3′UTR of each human gene obtained from the TargetScanS web server [40], [41], [42] (http://www.targetscan.org/).

For *COMT*, 604 bps downstream of the CDS (details can be accessed at http://idm.fudan.edu.cn/Apanda/) was determined as the 3′UTR of the *COMT* gene. The secondary structure of this sequence was predicted by Mfold with default settings [43] and the best predicted structure was selected (Figure S2).

For *MAOA*, the length of 3′UTR of human *MAOA* gene is 2,307 bps in the annotation of TargetScanS and the coding region of *MAOA* gene is on the negative strand. Therefore, a 4 kb sequence on positive strand upstream of the *MAOA* gene was selected, and the reverse complimentary sequences were analyzed by the same method described above to predict the polyA site of *MAOA* gene in panda. This sequence was also compared with the multiple sequences alignment file of the 3′UTR of human *MAOA* gene obtained from the TargetScanS. Finally, a 2320 bps sequence (details can be accessed at http://idm.fudan.edu.cn/Apanda/) was determined as the 3′UTR of the *MAOA* gene in panda.

For the other 10 genes (*LEP, ALDH2, PNMT, MAOB, ADRA1D, MC4R, OPRD1, GRIA3, HTR3E, GRM7*), detailed information can be seen in our database (http://idm.fudan.edu.cn/Apanda/).

### MicroRNA Target Prediction in Panda

For the above 12 genes, the same protocol was used to predict potential panda miRNA target. In detail, we first obtained all the miRNAs that have at least one target site in the 3′UTR of each human gene by TargetScanS. Then, those highly conserved miRNAs were filtered out by the following criteria: (i) miRNA should be present in at least six species; and (ii) only allow one mismatch in 22 nucleotides in all species. MiRNA sequences were downloaded from miRBase [44] (February, 2010). For those 12 genes, potential target sites on the 3′UTR of the filtered panda miRNAs were predicted by RNA22 server [45]. To ensure accuracy and sensitivity, we changed two parameter settings: (i) seed/nucleus is changed from 6 (default setting) to 7; and (ii) maximum folding energy for heteroduplex (Kcal/mol) is changed from −25 (default setting) to −20. The positive results for 12 genes were marked with “+” in table S3.

## Supporting Information

Figure S1 **The simulated panda and dog MAOA protein structure compared with human MAOA.** The panda or dog proteins were indicated with pink color for backbone and red color for special amino acid residues. Human proteins were indicated with blue color for backbone and yellow color for special amino acid residues. (**A**) The simulated panda MAOA compared with human MAOA. (**B**) The simulated dog MAOA compared with human MAOA.
(TIF)

Figure S2 **The secondary structure of 3′- UTR of gene** ***COMT*** **predicted by Mfold**. The part in red box was enlarged and put into Figure 3.
(TIF)

Figure S3 **A screen-shot of our giant panda genome analysis database.** (http://idm.fudan.edu.cn/Apanda/).
(TIF)

Table S1 **List of the 166 genes' full name and Ensembl number.** Empty box indicated that there is no ortholog found in that species.
(XLS)

Table S2 **Kozak sequence pattern of 81 genes in panda and human**. The genes have the same pattern at −3 and +4 position. A “y” is marked to indicate them matching the consensus sequence.
(DOC)

Table S3 **Kozak sequence pattern of 7 genes in all nine species.** The genes have different pattern at -3 and/or +4 position between human and panda. A “Y” is marked to indicate them matching the consensus sequence. An “X” is marked to indicate no ortholog identified in that species. A “-” is marked to indicate no “AUG” start codon in that species.
(DOC)

Table S4 **Predicted miRNAs for those 12 genes screened out by first two methods.** MiRNAs for those genes were predicted by RNA22 and there were no predicted miRNA for *PNMT*, *GRIA3* and *MC4R*.
(DOC)
