# Supplementary figures and images for: Why Does the Giant Panda Eat Bamboo? A Comparative Analysis of Appetite-Reward-Related Genes among Mammals
Source: PLoS One. 2011 Jul 27;6(7):e22602. doi: 10.1371/journal.pone.0022602 (PMC3144909; doi:10.1371/journal.pone.0022602)

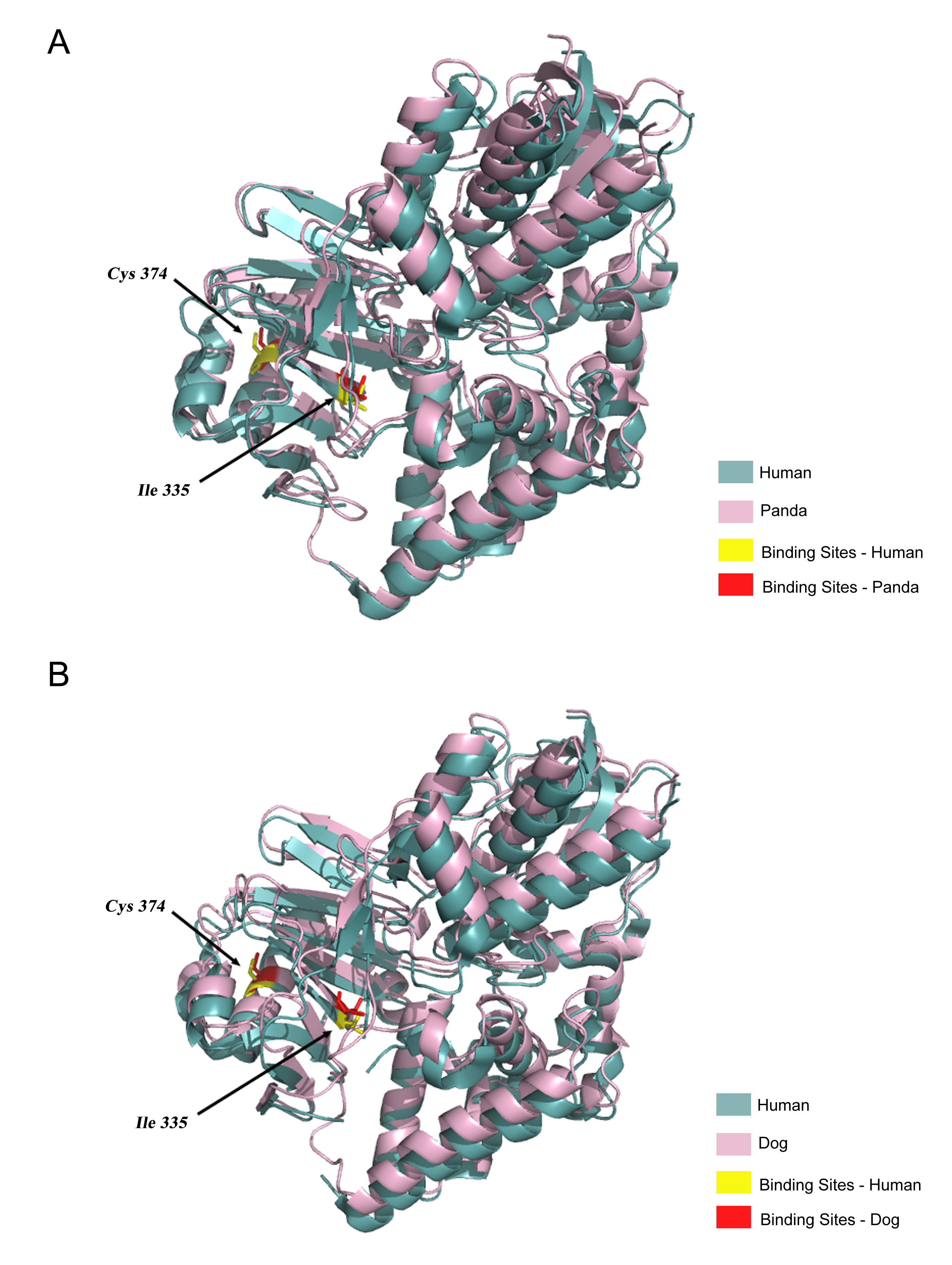

Supplement: Figure S1 — The simulated panda and dog MAOA protein structure compared with human MAOA. The panda or dog proteins were indicated with pink color for backbone and red color for special amino acid residues. Human proteins were indicated with blue color for backbone and yellow color for special amino acid residues. (A) The simulated panda MAOA compared with human MAOA. (B) The simulated dog MAOA compared with human MAOA. (TIF) [file pone.0022602.s001.tif]

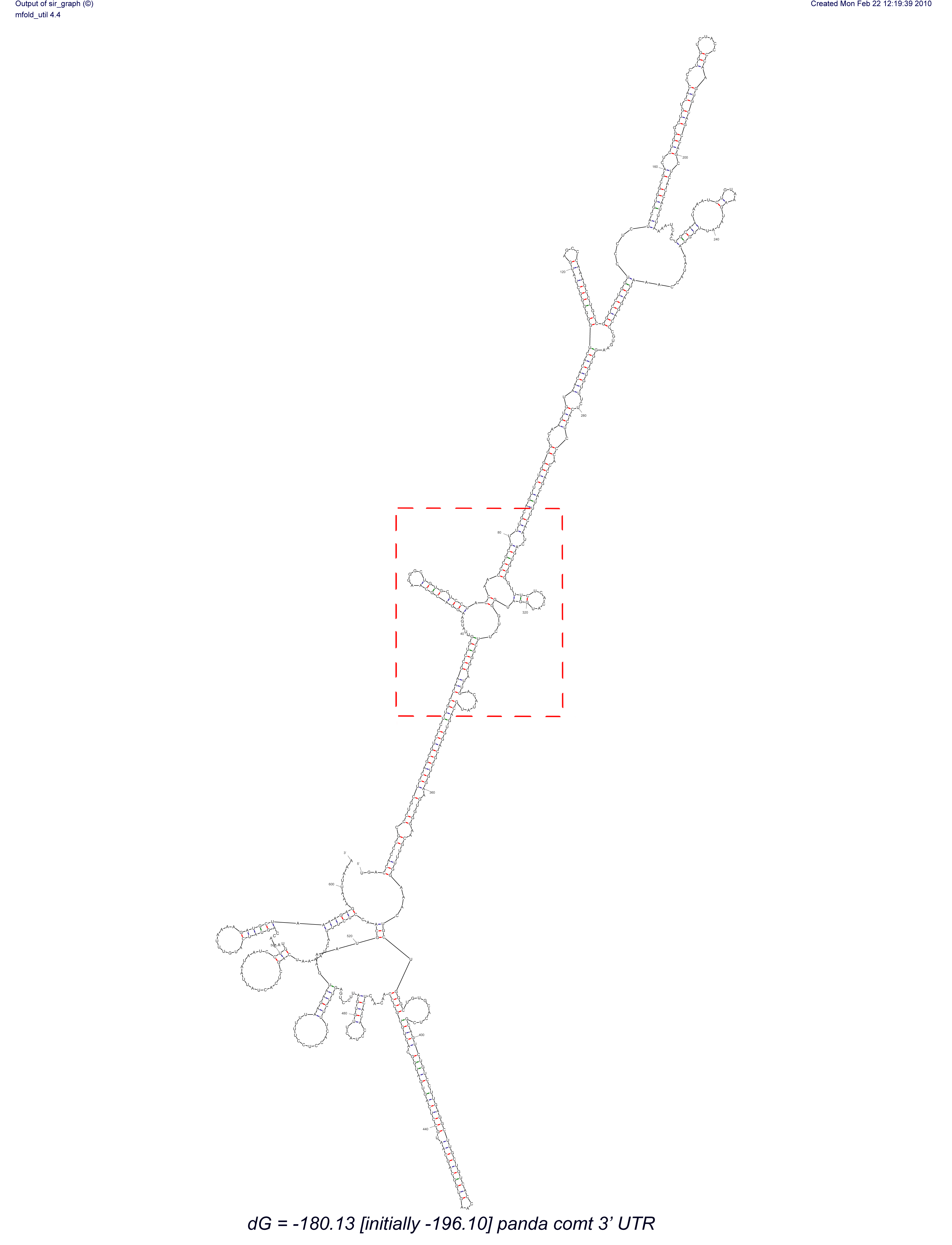

Supplement: Figure S2 — The secondary structure of 3′- UTR of gene COMT predicted by Mfold. The part in red box was enlarged and put into Figure 3. (TIF) [file pone.0022602.s002.tif]

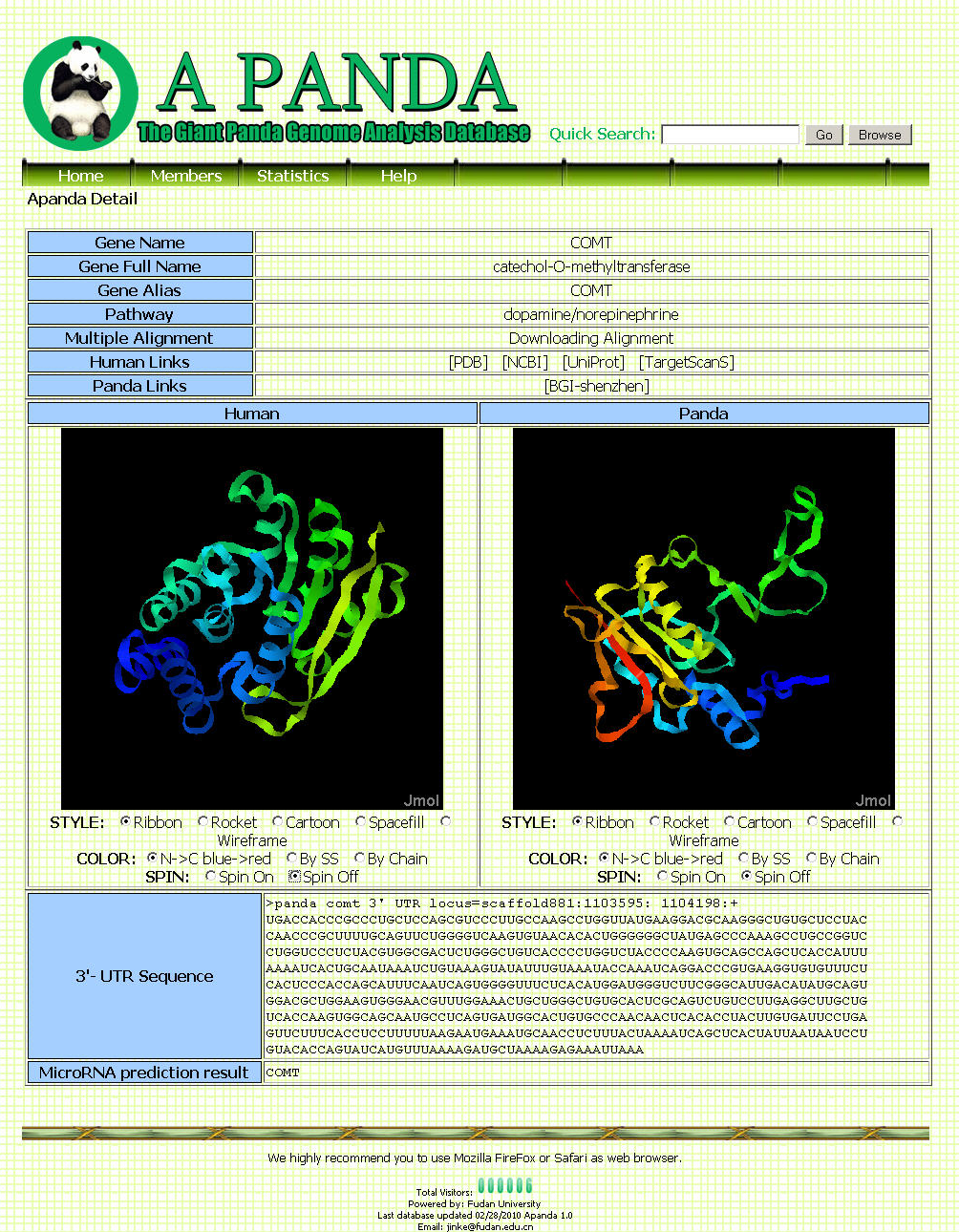

Supplement: Figure S3 — A screen-shot of our giant panda genome analysis database. (http://idm.fudan.edu.cn/Apanda/). (TIF) [file pone.0022602.s003.tif]
