# Supplementary material for: Why Does the Giant Panda Eat Bamboo? A Comparative Analysis of Appetite-Reward-Related Genes among Mammals
Source: PLoS One. 2011 Jul 27;6(7):e22602. doi: 10.1371/journal.pone.0022602 (PMC3144909; doi:10.1371/journal.pone.0022602)
Supplement: Table S2 — Kozak sequence pattern of 81 genes in panda and human. The genes have the same pattern at −3 and +4 position. A “y” is marked to indicate them matching the consensus sequence. (DOC) [file pone.0022602.s005.doc]

**Table S2** Kozak sequence pattern of 81 genes. The genes have the same pattern at -3 and +4 position. A “Y” is marked to indicate them matching the consensus sequence.

|  | Panda | | | Human | | |
| --- | --- | --- | --- | --- | --- | --- |
| Gene name | ATG | G+4 | R-3 | ATG | G+4 | R-3 |
| *GRIN1* | Y |  |  | Y |  |  |
| *AANAT* | Y |  | Y | Y |  | Y |
| *ADRA1B* | Y |  | Y | Y |  | Y |
| *ADRB2* | Y | Y | Y | Y | Y | Y |
| *ADRB3* | Y | Y | Y | Y | Y | Y |
| *ALDH16A1* | Y | Y | Y | Y | Y | Y |
| *ALDH18A1* | Y |  | Y | Y |  | Y |
| *ALDH1A2* | Y |  | Y | Y |  | Y |
| *ALDH1B1* | Y |  | Y | Y |  | Y |
| *ALDH1L1* | Y |  | Y | Y |  | Y |
| *ALDH1L2* | Y |  | Y | Y |  | Y |
| *ALDH3A1* | Y |  | Y | Y |  | Y |
| *ALDH3A2* | Y | Y | Y | Y | Y | Y |
| *ALDH8A1* | Y | Y | Y | Y | Y | Y |
| *ARRB1* | Y | Y | Y | Y | Y | Y |
| *ARRB2* | Y | Y | Y | Y | Y | Y |
| *CNR1* | Y |  | Y | Y |  | Y |
| *CREB* | Y |  |  | Y |  |  |
| *CRHR2* | Y | Y | Y | Y | Y | Y |
| *DDC* | Y |  | Y | Y |  | Y |
| *DRD1* | Y |  | Y | Y |  | Y |
| *DRD3* | Y | Y | Y | Y | Y | Y |
| *ESR2* | Y | Y | Y | Y | Y | Y |
| *GABRA1* | Y |  | Y | Y |  | Y |
| *GABRA2* | Y |  | Y | Y |  | Y |
| *GABRA3* | Y |  | Y | Y |  | Y |
| *GABRA4* | Y | Y | Y | Y | Y | Y |
| *GABRA5* | Y | Y | Y | Y | Y | Y |
| *GABRA6* | Y | Y | Y | Y | Y | Y |
| *GABRB1* | Y |  | Y | Y |  | Y |
| *GABRB2* | Y |  | Y | Y |  | Y |
| *GABRG1* | Y | Y |  | Y | Y |  |
| *GABRG2* | Y |  | Y | Y |  | Y |
| *GABRP* | Y |  | Y | Y |  | Y |
| *GABRQ* | Y | Y | Y | Y | Y | Y |
| *GCR* | Y | Y |  | Y | Y |  |
| *GHRELIN* | Y |  | Y | Y |  | Y |
| *GRIA1* | Y |  | Y | Y |  | Y |
| *GRIA4* | Y |  | Y | Y |  | Y |
| *GRIK4* | Y |  | Y | Y |  | Y |
| *GRIN2B* | Y |  | Y | Y |  | Y |
| *GRM1* | Y | Y | Y | Y | Y | Y |
| *GRM2* | Y | Y | Y | Y | Y | Y |
| *GRM3* | Y |  |  | Y |  |  |
| *GRM4* | Y |  | Y | Y |  | Y |
| *GRM8* | Y | Y | Y | Y | Y | Y |
| *HCRTR2* | Y |  | Y | Y |  | Y |
| *HRH1* | Y |  |  | Y |  |  |
| *HRH2* | Y | Y | Y | Y | Y | Y |
| *HRH4* | Y |  | Y | Y |  | Y |
| *HTR1A* | Y | Y | Y | Y | Y | Y |
| *HTR1B* | Y | Y | Y | Y | Y | Y |
| *HTR1D* | Y |  | Y | Y |  | Y |
| *HTR1F* | Y | Y | Y | Y | Y | Y |
| *HTR2B* | Y | Y |  | Y | Y |  |
| *HTR2C* | Y | Y | Y | Y | Y | Y |
| *HTR3C* | Y | Y | Y | Y | Y | Y |
| *HTR5A* | Y | Y | Y | Y | Y | Y |
| *HTR6* | Y | Y |  | Y | Y |  |
| *IL1RN* | Y | Y | Y | Y | Y | Y |
| *LEP* | Y |  | Y | Y |  | Y |
| *LEPR* | Y |  | Y | Y |  | Y |
| *LTA* | Y |  |  | Y |  |  |
| *MAOA* | Y | Y | Y | Y | Y | Y |
| *MCH* | Y | Y | Y | Y | Y | Y |
| *MCHR1* | Y |  | Y | Y |  | Y |
| *MCHR2* | Y |  | Y | Y |  | Y |
| *MTHFR* | Y | Y | Y | Y | Y | Y |
| *NOTCH4* | Y |  | Y | Y |  | Y |
| *NTRK2* | Y |  | Y | Y |  | Y |
| *OPRK1* | Y | Y | Y | Y | Y | Y |
| *PAH* | Y |  | Y | Y |  | Y |
| *PDYN* | Y | Y | Y | Y | Y | Y |
| *SLC18A1* | Y |  | Y | Y |  | Y |
| *SLC6A2* | Y |  |  | Y |  |  |
| *TH* | Y |  | Y | Y |  | Y |
| *TPH2* | Y |  |  | Y |  |  |
| *GABRR1* | Y |  |  | Y |  |  |
| *ADRA2A* | Y |  |  | Y |  |  |
| *NPY* | Y |  |  | Y |  |  |
| *GRIA2* | Y |  | Y | Y |  | Y |
