# Supplementary material for: Why Does the Giant Panda Eat Bamboo? A Comparative Analysis of Appetite-Reward-Related Genes among Mammals
Source: PLoS One. 2011 Jul 27;6(7):e22602. doi: 10.1371/journal.pone.0022602 (PMC3144909; doi:10.1371/journal.pone.0022602)
Supplement: Table S3 — Kozak sequence pattern of 7 genes in all nine species. The genes have different pattern at -3 and/or +4 position between human and panda. A “Y” is marked to indicate them matching the consensus sequence. An “X” is marked to indicate no ortholog identified in that species. A “-” is marked to indicate no “AUG” start codon in that species. (DOC) [file pone.0022602.s006.doc]

**Table S3.** Kozak sequence pattern of the 7 genes for all nine species. The genes have different pattern at -3 and/or +4 position between panda and human. A “y” is marked to indicate them matching the consensus sequence. A “X” is marked to indicate no ortholog identified in that species. A “-” is marked to indicate no “AUG” start codon in that species.

|  |  | ***MC4R*** | ***OPRD1*** | ***COMT*** | ***ADRA1D*** | ***GRIA3*** | ***HTR3E*** | ***GRM7*** |
| --- | --- | --- | --- | --- | --- | --- | --- | --- |
| Human | ATG | Y | Y | Y | Y | Y | Y | Y |
|  | G+4 | Y | Y |  |  | Y |  | Y |
|  | R-3 | Y | Y | Y | Y |  |  | Y |
|  | Strength | S | S | A | A | A | W | S |
| Panda | ATG | Y | Y | Y | Y | Y | Y | Y |
|  | G+4 |  | Y |  | Y | Y | Y |  |
|  | R-3 | Y |  |  | Y | Y | Y | Y |
|  | Strength | A | A | W | S | S | S | A |
| Chimpanzee | ATG | Y | Y | Y | Y | Y | Y | Y |
|  | G+4 | Y | Y |  |  | Y |  | Y |
|  | R-3 | Y | Y | Y | Y | Y |  | Y |
|  | Strength | S | S | A | A | S | W | S |
| Mouse | ATG | Y | Y | Y | Y | Y | X | Y |
|  | G+4 |  | Y |  |  | Y |  | Y |
|  | R-3 | Y | Y | Y | Y | Y |  | Y |
|  | Strength | A | S | A | A | S |  | S |
| Rattus | ATG | Y | Y | Y | Y | Y | X | X |
|  | G+4 |  | Y |  |  | Y |  |  |
|  | R-3 | Y | Y |  | Y | Y |  |  |
|  | Strength | A | S | W | A | S |  |  |
| Cow | ATG | Y | Y | Y | Y | Y | - | X |
|  | G+4 |  | Y |  |  |  |  |  |
|  | R-3 | Y | Y |  | Y |  |  |  |
|  | Strength | A | S | W | A | W |  |  |
| Horse | ATG | Y | Y | Y | - | Y | Y | Y |
|  | G+4 | Y | Y | Y | Y | Y | Y | Y |
|  | R-3 | Y | Y |  | Y | Y | Y | Y |
|  | Strength | S | S | A | A | S | S | S |
| Dog | ATG | Y | Y | Y | - | Y | - | - |
|  | G+4 |  | Y | Y | Y | Y |  | Y |
|  | R-3 | Y | Y |  | Y | Y | Y |  |
|  | Strength | A | S | A | A | S | W | W |
| Cat | ATG | - | Y | Y | X | Y | Y | - |
|  | G+4 |  | Y |  |  |  | Y |  |
|  | R-3 |  | Y |  |  | Y | Y |  |
|  | Strength |  | S | W |  | A | S |  |
