# Supplementary material for: Why Does the Giant Panda Eat Bamboo? A Comparative Analysis of Appetite-Reward-Related Genes among Mammals
Source: PLoS One. 2011 Jul 27;6(7):e22602. doi: 10.1371/journal.pone.0022602 (PMC3144909; doi:10.1371/journal.pone.0022602)
Supplement: Table S4 — Predicted miRNAs for those 12 genes screened out by first two methods. MiRNAs for those genes were predicted by RNA22 and there were no predicted miRNA for PNMT, GRIA3 and MC4R. (DOC) [file pone.0022602.s007.doc]

**Table S4.** Predicted miRNAs for those 12 genes screened out by first two methods. MiRNAs for those genes were predicted by RNA22 and there were no predicted miRNA for *PNMT*, *GRIA3*, and *MC4R*.

| **Genes** | | **miRNA family** | **Query miRNA** | **human** | **panda** |
| --- | --- | --- | --- | --- | --- |
| *COMT* | | miR-186 | hsa-miR-186 | **－** | **－** |
|  |  | miR-199a-5p | hsa-miR-199a-5p | **－** | **＋** |
|  |  | miR-214 | hsa-miR-214 | **－** | **－** |
|  |  | miR-218 | hsa-miR-218 | **－** | **－** |
|  |  | miR-410 | hsa-miR-410 | **－** | **－** |
|  |  | miR-433 | hsa-miR-433 | **－** | **－** |
| *MAOA* | | miR-22 | hsa-miR22 | **＋** | **－** |
|  |  | miR-34a | hsa-miR34a | **＋** | **－** |
|  |  | miR-122 | hsa-miR122 | **＋** | **＋** |
|  |  | miR-132 | hsa-miR132 | **＋** | **－** |
|  |  | miR-148a | hsa-miR148a | **＋** | **－** |
|  |  | miR-150 | hsa-miR150 | **－** | **＋** |
|  |  | miR-152 | hsa-miR152 | **＋** | **－** |
|  |  | miR-181a | hsa-miR181a | **＋** | **－** |
|  |  | miR-181d | hsa-miR181d | **＋** | **＋** |
|  |  | miR-187 | hsa-miR187 | **＋** | **＋** |
|  |  | miR-196a | hsa-miR196a | **＋** | **＋** |
|  |  | miR-196b | hsa-miR196b | **＋** | **＋** |
|  |  | miR-197 | hsa-miR197 | **－** | **－** |
|  |  | miR-205 | hsa-miR205 | **－** | **－** |
|  |  | miR-223 | hsa-miR223 | **＋** | **－** |
|  |  | miR-326 | hsa-miR326 | **－** | **－** |
|  |  | miR-378 | hsa-miR378 | **＋** | **－** |
|  |  | miR-410 | hsa-miR410 | **－** | **－** |
|  |  | miR-448 | hsa-miR448 | **＋** | **＋** |
|  |  | miR-449a | hsa-miR449a | **＋** | **－** |
|  |  | miR-495 | hsa-miR495 | **－** | **－** |
|  |  | miR-539 | hsa-miR539 | **＋** | **＋** |
| *MAOB* | | miR-494 | hsa-miR-494 | **－** | **－** |
|  |  | miR-217 | hsa-miR-217 | **＋** | **＋** |
|  |  | miR-194 | hsa-miR-194 | **－** | **－** |
| *LEP* | miR-9 | | hsa-miR-9 | **＋** | **＋** |
| *ALDH2* | miR-30c | | hsa-miR-30c | **－** | **－** |
| *ADRA1D* | miR-30c | | hsa-miR-30c | **－** | **＋** |

| **Genes** | **miRNA family** | **Query miRNA** | **human** | **panda** |
| --- | --- | --- | --- | --- |
| *OPRD1* | miR-326 | hsa-miR-326 | **－** | **＋** |
|  | miR-184 | hsa-miR-184 | **＋** | **－** |
|  | miR-874 | hsa-miR-874 | **＋** | **－** |
| *HTR3E* | miR-873 | has-miR-873 | **－** | **－** |
|  | miR-9 | has-miR-9 | **－** | **＋** |
|  | miR-214 | has-miR-214 | **＋** | **－** |
|  | miR-383 | has-miR-383 | **－** | **－** |
|  | miR-539 | has-miR-539 | **＋** | **－** |
| *GRM7* | miR-449a | hsa-miR-449a | **＋** | **＋** |
|  | miR-34a | hsa-miR-34a | **＋** | **＋** |
